# Physiological Stress in Rescued Wild Koalas (*Phascolarctos cinereus*) Being Held in a Rehabilitation Sanctuary: A Pilot Study

**DOI:** 10.3390/ani11102864

**Published:** 2021-09-30

**Authors:** Renae Charalambous, Troy Simonato, Matthew Peel, Edward J. Narayan

**Affiliations:** 1 School of Agriculture and Food Sciences, Faculty of Science, The University of Queensland, St Lucia, Brisbane, QLD 4072, Australia; r.charalambous@uq.edu.au; 2 School of Science, Western Sydney University, Locked Bag 1797, Penrith, NSW 2751, Australia; t.simonato@westersydney.edu.au (T.S.); m.peel@westernsydne.edua.au (M.P.); 3 Queensland Alliance for Agriculture and Food Innovation, The University of Queensland, St Lucia, Brisbane, QLD 4072, Australia

**Keywords:** faeces, fur, glucocorticoids, stress

## Abstract

**Simple Summary:**

Koalas (*Phascolarctos cinereus*) are one of Australia’s most charismatic native small marsupial species, but populations are rapidly declining throughout Australia as they continue to face increasing pressure from a changing ecosystem. In response to stressors, koalas will use their hypothalamic–pituitary–adrenal (HPA) axis to produce catecholamines and glucocorticoids (e.g., cortisol). In this pilot study, we set out to quantify faecal and fur cortisol metabolites in wild rescued koalas undergoing wildlife rehabilitation. Sampling occurred sporadically over four months (the start of September 2018 to the end of December 2018), and was performed on three rescued koalas (Maree, Tai, and Solstice) being held at the rehabilitation centre. Results of this study show that between the three koalas, the highest recorded faecal cortisol result was 241 ng/g, and the lowest recorded faecal cortisol result was 4 ng/g, whereas the highest recorded fur cortisol result was 1.75 ng/g, and the lowest recorded fur cortisol result was 0.10 ng/g. Statistically, there was a significant difference between all three koalas and their faecal cortisol responses, as well as their fur cortisol responses. Statistically for Maree and Solstice, there was a significant difference in their faecal cortisol response between days when a stressor was recorded, and days when a stressor was not recorded. However, statistically for Tai, this was not the case, as there was no significant difference in his faecal cortisol response between days when a stressor was recorded, and days when a stressor was not recorded. In summary, the hypothesis that faecal glucocorticoids and fur glucocorticoids between koalas will differ based on individual responses to stressors was true as a whole but individually, this hypothesis was true for Maree and Solstice, but untrue for Tai. Measuring faecal and fur glucocorticoids is the first step in understanding how koalas undergoing wildlife rehabilitation respond to stressors.

**Abstract:**

Koalas (*Phascolarctos cinereus*) are one of Australia’s most charismatic native small marsupial species. Unfortunately, populations of koalas are rapidly declining throughout Australia as they continue to face increasing pressure from a changing ecosystem. All wildlife species to some degree will use their hypothalamic–pituitary–adrenal (HPA) axis in response to stress. Depending on the duration of activation, the stress response can lead to either acute or chronic side effects and is modulated through the neuroendocrine stress system with the release of catecholamines and glucocorticoids (e.g., cortisol). It is well known that rehabilitation sanctuaries are inherently stressful for all animals, in particular for rescued wild koalas, as it is an unfamiliar environment where the animals cannot predict or control what will happen to them. In this pilot study, we set out to quantify faecal and fur cortisol metabolites in wild rescued koalas undergoing wildlife rehabilitation. Absolute levels of acute and chronic stress were indexed non-invasively, with faecal samples taken to evaluate acute stress, and fur samples taken to evaluate chronic stress. Sampling occurred sporadically over four months (the start of September 2018 to the end of December 2018), and was performed on three rescued koalas (Maree, Tai, and Solstice) being held at the rehabilitation centre. Results of this study show that between the three koalas, the highest recorded faecal cortisol result was 241 ng/g, and the lowest recorded faecal cortisol result was 4 ng/g, whereas the highest recorded fur cortisol result was 1.75 ng/g, and the lowest recorded fur cortisol result was 0.10 ng/g. Statistically, there was a significant difference between all three koalas and their faecal cortisol responses, as well as their fur cortisol responses. Statistically for Maree and Solstice, there was a significant difference in their faecal cortisol response between days when a stressor was recorded, and days when a stressor was not recorded. However, statistically for Tai, this was not the case, as there was no significant difference in his faecal cortisol response between days when a stressor was recorded, and days when a stressor was not recorded. In summary, the hypothesis that faecal glucocorticoids and fur glucocorticoids between koalas will differ based on individual responses to stressors was true as a whole, but individually, this hypothesis was true for Maree and Solstice, but untrue for Tai. The use of biological samples such as faeces and fur to obtain readings of glucocorticoids is a method of measuring absolute levels of physiological stress that is still evolving for koalas, and there is no current glucocorticoid baseline with which to compare the results of this study; although, measuring faecal and fur glucocorticoids is the first step in understanding how koalas undergoing wildlife rehabilitation respond to stressors.

## 1. Introduction

The koala (*Phascolarctos cinereus*) is one of the most charismatic native small marsupial species iconic to Australian identity [1]. Unfortunately, populations of koalas are rapidly declining throughout Australia, particularly in Queensland and New South Wales [2,3]. Threats faced by koalas are varied, but predominantly include trauma from vehicle collision, being attacked by animals (e.g., cats, dogs, cattle), and succumbing to disease [2,3]. A previous study of wild koalas admitted into clinical care in New South Wales indicate that between 1989 and 2018, 9.7% of koalas were struck by a motor vehicle, 4.4% sustained an injury from another animal, and 34.4% were diagnosed with a disease [2]. Similarly, in Queensland between 1997 and 2013, 15.5% of koalas were struck by a motor vehicle, 5.2% sustained injury from another animal, and 55.6% were diagnosed with a disease [3].

The goal of wildlife rehabilitation is the successful transition and return of an individual back to its natural habitat, in a fit and healthy state to reproduce and perform all other natural functions and behaviours that benefit the environment and the species [4]. However, in reference to that earlier study of wild koalas admitted into clinical care, in New South Wales, only 20.7% were released back into the wild [2], and only 17.2% were released back into the wild in Queensland [3]. These results are echoed by other studies, where in a study of 362,058 animal rescues in New South Wales, 44.8% of wildlife survived, and were assumed to go on to be released back into the wild [5]. Despite so many wild animals being admitted into clinical care, the effectiveness of wildlife rehabilitation as a conservation strategy remains unclear [4]. The major constraint identified as a barrier to treating wildlife in clinical care is a lack of knowledge/skills and time, although cost was also an identified constraint [6].

Stress is described as an unpredictable and/or uncontrollable stimulus which elicits a physiological response [7]. This response starts with activation of the hypothalamus–pituitary–adrenal (HPA) axis which signals the hypothalamus to synthesise corticotrophin-releasing factor (CRF) neuropeptides to stimulate the pituitary gland to release adrenocorticotropic hormone (ACTH) [8]. The result of this is a secretion of glucocorticoids which aid in the production of sugars aimed at providing energy to either ‘fight or flight’ from the said stressor [8]. The neurogenic adrenomedullary response through secretion of catecholamines from the chromaffin cells of the adrenal medulla provides the initial ‘fight or flight’ response, or the first-wave response occurring within seconds while glucocorticoids act as part of the slower wave which occurs over the course of minutes [9]. The hormonal responses bring about necessary physiological changes that enable the animal to cope with the stressor such as the diversion of energy to exercising muscles, immune response, decreased feeding and sharpened cognition, etc. [9]. Once the stimulus causing stress has ceased, the process of homeostasis acts to return the body to a pre-stress state optimal to maintain life, and the animal is said to have experienced an acute stressor [10]. In the event that the stressor does not cease and the body can no longer maintain homeostasis, the animal is said to be experiencing chronic stress [11]. Stimuli causing stress can stem from external factors (e.g., a loud noise) or internal factors (e.g., dehydration), meaning a single event can impact individuals differently [12].

It is well documented that chronic stress can have deleterious effects on physiological health and often leads to a greater susceptibility to disease [9]. This is because stress is adaptive for an animal over the short term as present energy use is prioritised over future energy storage [13]. During activation of the HPA-axis and the production of glucocorticoids, the functions of immunological processes are altered, which changes immune gene expression on target tissues, having complex effects on both innate and acquired immunity [14]. For example, glucocorticoids reduce the trafficking of leukocytes and accessory immune cells (cells which are responsible for fighting infection), as well as suppressing the secretion of proinflammatory cytokines (regulators of inflammation as a response to infection to heal and repair) [15]. The pathogenesis of chronic stress related disorders can be explained by sustained, excessive secretion and effects of the major mediators of stress and sickness syndromes, which influence the activities of multiple homeostatic systems [16,17]. These disorders thus represent chronic, maladaptive effects of two physiological processes whose mediators are meant to be secreted in a quantity-limited and time-limited fashion but have gone awry [15]. Koalas exposed to chronic stress are at risk of immune cell related disorders including but not limited to, inflamed tissues, systemic infection, and organ dysfunction [18].

Faeces are a common biological sample used to obtain readings of glucocorticoids [19]. The use of faecal sampling is due to the fact that it is almost a completely non-invasive procedure that is able to be performed by untrained personnel [20]. Faeces are able to be collected fairly easily from animals within the field after careful observation of recent defecation [21]. The only concern however, is that unlike other biological samples such as fur, right after collection, faeces need to be stored at −20 °C [22]. Following collection and appropriate storage, a cortisol based enzyme-immunoassay can be used to index glucocorticoid hormones [22]. A previous study discovered excretory lag-times of glucocorticoid hormones between koala sexes with 24 h for females, and 48 h for males [23]. This is due to the excessively long gut system of the species, as well as natural fluctuations in reproductive hormones leading to increased metabolic demands [24,25]. Because koalas have a lengthy gut with a special fibre-digesting caecum, it can take several days before hormone metabolism occurs, and excretion of steroidal end-products takes place [23]. Therefore, studies using minimally invasive hormone monitoring techniques may need a more frequent sampling regime (e.g., up to 10 days) [23].

Like faeces, fur too is a common biological sample used to obtain readings of glucocorticoids [26]. Fur collection is also an almost completely non-invasive procedure, and can be collected from animals without capturing them such as through the use of hair traps [27]. Alternatively, fur can be shaved when an animal is undergoing routine medical checks, removing the need for additional capture and handling [28]. Furthermore, fur is easy to store as it can be sealed in paper envelopes or aluminium foil, and kept at ambient temperature away from direct sunlight [29,30]. Following collection and appropriate storage, a cortisol based enzyme-immunoassay can be used to index glucocorticoid hormones [28]. Measurements of glucocorticoids in fur indicate an average cortisol concentration over a period of weeks to months, as the predictable rate of hair growth is ~1 cm per month, and blood-borne hormones such as glucocorticoids are known to be incorporated into fur during the active growth phase [26,29,31].

Most recently, Santamaria et al. have determined the exact glucocorticoid metabolite present in koala faeces using liquid-chromatography and mass spectroscopy [32]. Availability of group specific assay kits are challenging, and the majority of wildlife studies have relied on commercially available kits (e.g., Cayman Chemical or Abor Assay). Whichever assay is chosen, or developed in-house and used, it is important to interpret the results with care accounting for intra- and inter-individual variation in hormone metabolism, as well as potential impacts of disease (e.g., effect of chlamydia on gut microflora), and environmental dynamics (e.g., consumption of hardy leaves in the dry season versus the wet season) [33]. These factors, plus several others, are critical for researchers performing assays [33].

The aim of this study was to quantify faecal and fur cortisol metabolites in wild rescued koalas undergoing wildlife rehabilitation. It was hypothesised that faecal glucocorticoids and fur glucocorticoids between koalas would differ based on individual responses to stressors.

## 2. Materials and Methods

Research was performed in accordance with relevant guidelines and regulations. Formal approval was granted by the Western Sydney University Animal Care and Ethics (ACEC) Committee (approval number: A12373).

### 2.1. Study Site

This research was performed in collaboration with the Port Stephens Koala Hospital, which is located at 562 Gan Gan Road, One Mile (GPS Coordinates: −32.763792, 152.115904). Koalas are admitted into the care of the Port Stephens Koala Hospital as they operate a fully functional rehabilitation sanctuary for injured and orphaned koalas within the New South Wales region.

### 2.2. Koalas

The three koalas (Maree, Tai, and Solstice) within this pilot study were chosen based on whichever was currently in care at the Port Stephens Koala Hospital during the period of data collection (September 2018 to December 2018).

Maree is a female, young adult who was admitted into care at the Port Stephens Koala Hospital in November 2017 after being struck by a motor vehicle. The injuries experienced by Maree rendered her permanently blind, thus unable to be released back into the wild. During the period of data collection, Maree was being held at the Port Stephens Koala Hospital while an application was being processed for her to remain in permanent care at a rehabilitation sanctuary.

Tai is a male, young adult who was admitted into care at the Port Stephens Koala Hospital in October 2017 after being found sitting on the ground by a member of the public. Tai was found to have bilateral cataracts and nystagmus, which required extensive veterinary intervention. During the period of data collection, Tai was being held at the Port Stephens Koala Hospital where his condition was being monitored by veterinarians.

Solstice is a male, young adult who was admitted into care at the Port Stephens Koala Hospital in June 2018 after being struck by a motor vehicle. The injuries experienced by Solstice included fractures to his elbow, eye socket and jaw. During the period of data collection, Solstice moved between home care and being held at the Port Stephens Koala Hospital where his condition was monitored by veterinarians.

### 2.3. Data Collection

Faecal samples were collected almost daily during routine husbandry activities (conducted in the morning) by staff from the enclosures of the three koalas (Maree, Tai, and Solstice). Fur samples were collected opportunistically by veterinarians from the three koalas (Maree, Tai, and Solstice). When each sample was collected, it was stored in a labelled resealable bag (name of koala and date collected) and stored in a freezer (−18 °C) before being transported on ice to the laboratory for analysis. During delivery and analysis of the samples, the faeces were kept frozen to minimise effects of deterioration.

Furthermore, stressors were observed and recorded by staff at the Port Stephens Koala Hospital and were verified by the koala manager on site each day. All stressors were categorized as external and refer to any noxious environmental stimulus that generates physical distress to one or more of the koalas in the sanctuary. A comprehensive understanding of these stressors is listed in Table 1.

### 2.4. Glucocorticoid Extraction

#### Faeces and fur

Once removed from the freezer, each faeces or fur sample was dehydrated in a freeze dryer until they were completely dry. Each sample was then individually ground into a fine powder using a mortar and pestle, which was cleaned between samples using 10% ethanol. Each sample was then sifted through a fine mesh strainer to remove any course particles.

For faecal sample extraction, two grams (g) of the ground and sifted sample was placed in a labelled test tube with 2 millilitres (mL) of 90% ethanol solution. On medium-high speed, the test tubes were vortexed in an Eppendorf Mini-spin centrifuge for 30 s to mix the solution, and then placed in an 80 °C water bath for 10 min. While in the water bath, the test tubes were gently shaken to ensure the samples remained submerged in the ethanol. The contents of the test tubes were then poured into la-belled Eppendorf tubes, closed, then centrifuged at 10,000× *g* revolutions per minute (RPM) for 5 min. At this stage, the liquid residue should have separated from the hormones dissolved in the ethanol, and 0.6 mL of solution was aliquoted into a new and clean labelled Eppendorf tube. Left open, the tubes were stored in a laminar flow chamber for 24 h, ensuring enough time for the ethanol to completely evaporate, then 1 mL of assay buffer was added. The tubes were vortexed at medium-high speed in an Eppendorf Mini-spin centrifuge for 30 s, and then centrifuged at 10,000× *g* force for 10 min. Following this, 850 microliters (µL) of supernatant was pipetted into a new and clean labelled Eppendorf tube, ensuring any of the solid section of the solution was avoided. Note: if the sample looked cloudy, tubes were re-centrifuged for 10 min and pipetted into a new and clean labelled Eppendorf tube.

For fur sample extraction, 60 milligrams (g) of the ground and sifted sample was placed in a labelled Eppendorff tube with 1 millilitre (mL) of 90% methanol solution. The tubes were left in a fridge overnight (4 °C). The next morning, the Eppendorff tubes were vortexed with lid-closed in an Eppendorf Mini-spin centrifuge for 30 s to mix the solution, and then placed in a laminar flow chamber for 24 h, ensuring enough time for the methanol to completely evaporate. 1 mL of assay buffer was added was added to each tube ready for assay.

### 2.5. Hormone Analysis

Glucocorticoid concentrations for both faecal and fur extract were determined using a polyclonal anti-cortisol antiserum diluted to 1:15,000, horseradish peroxidase (HRP) conjugated cortisol 1:80,000, and cortisol standards (1.56–400 pg well^−1^). Sample extracts were then assayed in duplicate on Nunc Maxisorp™ plates (96 wells) (Sigma Aldrich, Sydney, Australia). Plates were coated with diluted cortisol antibody and left to stand and incubate for a minimum 12 h in a fridge at 4 °C. The plates were washed using an automated plate washer (ELx50, BioTek™, Sursee, Switzerland). The dilution factor for the glucocorticoids in koala faeces and fur samples were based on the concentration of pooled samples that resulted in 50% binding on the parallelism curve (as seen in [23,28]). For each assay, 50 µL of cortisol standard, control, and diluted faecal or fur extract was added to each well based on the plate map, immediately following 50 µL of HRP. Plates were covered and incubated at room temperature for 2 h, following a wash and 50 µL of substrate buffer to generate a colour change. Colour reaction was halted after 15 min using 50 µL of stop solution, and the plates were read at 450 nanometres (nm) on an ELx800 (BioTek™, Sursee, Switzerland) microplate reader.

### 2.6. Data Analysis

Data analysis was performed in Microsoft Excel 2021^©^ (version 2103) using a Single Factor ANOVA to compare the level of significant difference in hormone levels between periods (stressor versus no stressor recorded by sampling days) and compared mean hormone levels between the three individual koalas. See supplementary file 1.0 for all primary data and test results. Data was checked for homogeneity of variance prior to performance of the statistical test. Hormone analysis yielded glucocorticoid results represented as cortisol nanogram per gram (ng/g). Faecal glucocorticoid results over the period of data collection for all three koalas were then graphed individually and together as a box and whisker plot. Fur glucocorticoid results over the period of data collection for all three koalas were then graphed together as a box and whisker plot. Any outliers were then removed from the dataset of all five graphs.

## 3. Results

**Figure 1:**
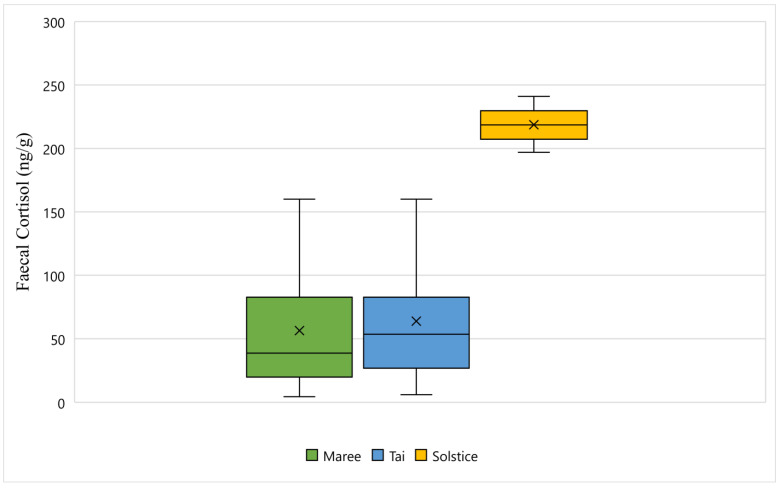
This figure displays the faecal cortisol response for Maree, Tai, and Solstice while undergoing wildlife rehabilitation from the start of September 2018 to the end of December 2018 (N = 120, *p*-Value = 0.000). Maree, Tai, and Solstice are represented as green, blue, and yellow respectively, and the cross represents the average faecal cortisol reading for each koala.

Figure 1 displays the faecal cortisol response for Maree, Tai, and Solstice while undergoing wildlife rehabilitation from the start of September 2018 to the end of December 2018. The average faecal cortisol result for Maree and Tai was 56 ng/g and 63 ng/g respectively, however the average for Solstice was 218 ng/g (Figure 1). The bottom whisker for Maree and Tai measured 4 ng/g and 5 ng/g respectively, whereas the bottom whisker for Solstice measured 197 ng/g (Figure 1). Furthermore, the top whisker for Maree and Tai both measured 160 ng/g, however for Solstice, the top whisker measured 241 ng/g (Figure 1). Statistically, there is a significant difference between all three koalas and their faecal cortisol response (*p*-Value < 0.05) (Figure 1).

**Figure 2:**
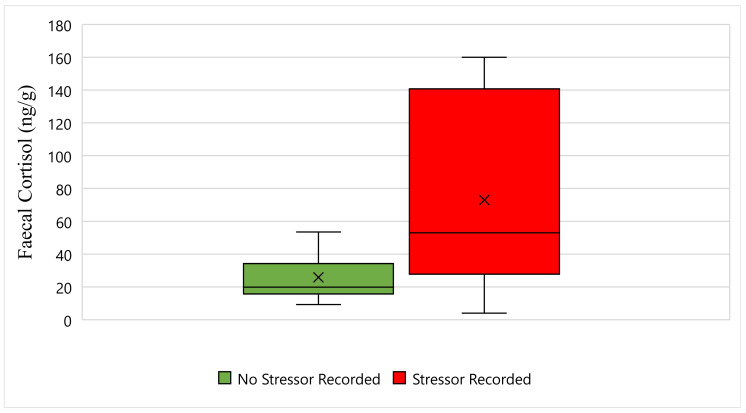
This figure displays the faecal cortisol response for Maree while undergoing wildlife rehabilitation from the start of September 2018 to the end of December 2018 (N = 29, *p*-Value = 0.001). Days where no stressor was recorded are represented as green, the days where a stressor was recorded are represented as red, and the cross represents the average faecal cortisol reading for each category.

Figure 2 displays the faecal cortisol response for Maree while undergoing wildlife rehabilitation from the start of September 2018 to the end of December 2018. The average faecal cortisol result for Maree on a day where no stressor was recorded was 25 ng/g, whereas the average faecal cortisol result on a day where a stressor was recorded was 73 ng/g (Figure 2). The bottom whisker for Maree on a day where no stressor was recorded measured 9 ng/g, whereas the bottom whisker on a day where a stressor was recorded measured 4 ng/g (Figure 2). Furthermore, the top whisker for Maree on a day where no stressor was recorded measured 53 ng/g, whereas the top whisker on a day where a stressor was recorded measured 160 ng/g (Figure 2). Statistically, there is a significant difference in Maree’s faecal cortisol response between days when a stressor was recorded, and days when a stressor was not recorded (*p*-Value ≤ 0.05) (Figure 2).

**Figure 3:**
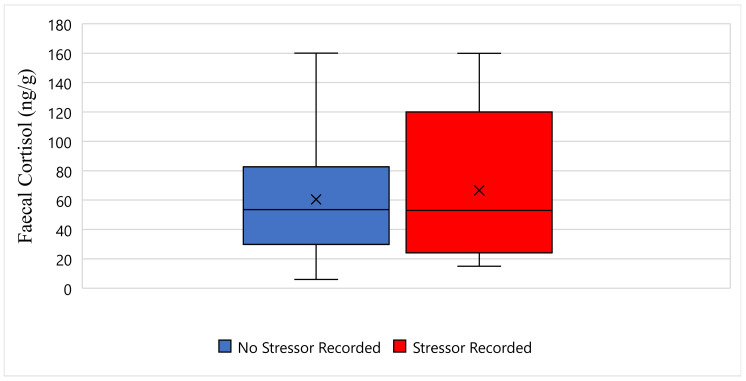
This figure displays the faecal cortisol response for Tai while undergoing wildlife rehabilitation from the start of September 2018 to the end of December 2018 (N = 44, *p*-Value = 0.915). Days where no stressor was recorded are represented as blue, the days where a stressor was recorded are represented as red, and the cross represents the average faecal cortisol reading for each category.

Figure 3 displays the faecal cortisol response for Tai while undergoing wildlife rehabilitation from the start of September 2018 to the end of December 2018. The average faecal cortisol result for Tai on a day where no stressor was recorded was 60 ng/g, whereas the average faecal cortisol result on a day where a stressor was recorded was 66 ng/g (Figure 3). The bottom whisker for Tai on a day where no stressor was recorded measured 5 ng/g, whereas the bottom whisker on a day where a stressor was recorded measured 15 ng/g (Figure 3). Furthermore, the top whisker for Tai on a day where no stressor was recorded, and on a day where a stressor was recorded, both measured 160 ng/g (Figure 3). Statistically, there is no significant difference in Tai’s faecal cortisol response between days when a stressor was recorded, and days when a stressor was not recorded (*p*-Value ≥ 0.05) (Figure 3).

**Figure 4:**
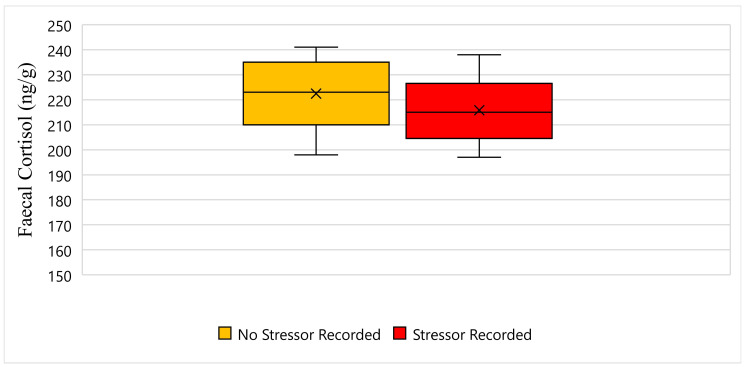
This figure displays the faecal cortisol response for Solstice while undergoing wildlife rehabilitation from the start of September 2018 to the end of December 2018 (N = 44, *p*-Value = 0.000). Days where no stressor was recorded are represented as yellow, the days where a stressor was recorded are represented as red, and the cross represents the average faecal cortisol reading for each category.

Figure 4 displays the faecal cortisol response for Solstice while undergoing wildlife rehabilitation from the start of September 2018 to the end of December 2018. The average faecal cortisol result for Solstice on a day where no stressor was recorded was 222 ng/g, whereas the average faecal cortisol result on a day where a stressor was recorded was 215 ng/g (Figure 4). The bottom whisker for Solstice on a day where no stressor was recorded measured 198 ng/g, and similarly, the bottom whisker on a day where a stressor was recorded measured 197 ng/g (Figure 4). Furthermore, the top whisker for Solstice on a day where no stressor was recorded measured 241 ng/g, whereas the top whisker on a day where a stressor was recorded measured 238 ng/g (Figure 4). Statistically, there is a significant difference in Solstice’s faecal cortisol response between days when a stressor was recorded, and days when a stressor was not recorded (*p*-Value ≤ 0.05) (Figure 4).

**Figure 5:**
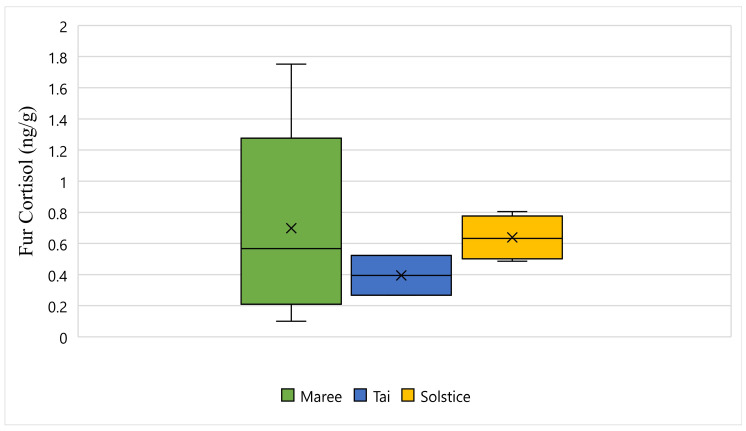
This figure displays the fur cortisol response for Maree, Tai, and Solstice while undergoing wildlife rehabilitation from the start of September 2018 to the end of December 2018 (N = 15, *p*-Value = 0.000). Maree, Tai, and Solstice are represented as green, blue and yellow respectively, and the cross represents the average fur cortisol reading for each koala.

Figure 5 displays the fur cortisol response for Maree, Tai, and Solstice while undergoing wildlife rehabilitation from the start of September 2018 to the end of December 2018. The average fur cortisol result for Maree was 0.69 ng/g, the average for Tai was 0.39 ng/g, and the average for Solstice was 0.63 ng/g (Figure 5). The bottom whisker for Maree measured 0.10 ng/g, the bottom whisker for Solstice measured 0.48 ng/g, and although there was no bottom whisker recorded, the minimum value was the same as the first quartile for Tai and measured 0.26 ng/g (Figure 5). Furthermore, the top whisker for Maree measured 1.75 ng/g, the top whisker for Solstice measured 0.80 ng/g, and although there was no top whisker recorded, the maximum value was the same as the third quartile for Tai and measured 0.52 ng/g (Figure 5). Statistically, there is a significant difference between all three koalas and their fur cortisol response (*p*-Value ≤ 0.05) (Figure 5).

## 4. Discussion

The aim of this study is to quantify faecal and fur cortisol metabolites in wild rescued koalas undergoing wildlife rehabilitation. It is hypothesised that faecal glucocorticoids and fur glucocorticoids between koalas will differ based on individual responses to stressors.

Results of this study show that between the three koalas, the highest recorded faecal cortisol result was 241 ng/g, and the lowest recorded faecal cortisol result was 4 ng/g, whereas the highest recorded fur cortisol result was 1.75 ng/g, and the lowest recorded fur cortisol result was 0.10 ng/g. Statistically, there was a significant difference between all three koalas and their faecal cortisol responses, as well as their fur cortisol responses. Statistically for Maree and Solstice, there was a significant difference in their faecal cortisol response between days when a stressor was recorded, and days when a stressor was not recorded. However, statistically for Tai, this was not the case, as there was no significant difference in his faecal cortisol response between days when a stressor was recorded, and days when a stressor was not recorded. In summary, the hypothesis that faecal glucocorticoids and fur glucocorticoids between koalas will differ based on individual responses to stressors is true as a whole, but individually, this hypothesis is true for Maree and Solstice, but untrue for Tai.

The use of biological samples such as faeces and fur to obtain readings of glucocorticoids is a method of measuring absolute levels of physiological stress that is still evolving for koalas. This means that there is no current glucocorticoid baseline with which to compare the results of this study. A previous study tested different enzyme immunoassays on faecal samples from 13 captive koalas over a 12-month period, which generated some important preliminary baseline data [34]. However, a gap remains for baseline glucocorticoid data for faecal and fur samples in wild koalas. As a result, measuring faecal and fur glucocorticoids is the first step in understanding how koalas undergoing wildlife rehabilitation respond to stressors experienced within clinical care.

It is well known that rehabilitation sanctuaries are inherently stressful for all animals, as it is an unfamiliar environment where the animal cannot predict or control what will happen to them [35,36]. This experience is even more challenging for wild animals than it is for domestic animals, as it is entirely unnatural for wild animals to be around humans [36]. During this study, a number of tourists set up camping sites adjacent to the Port Stephens Koala Hospital for Christmas/New Year’s celebrations. It was anticipated that this would be an additional stressor for Maree, Tai, and Solstice, as rehabilitation sanctuaries are unintentionally riddled with stressors [36], The various stressors experienced by the koalas in this study include bellowing, campers, fire-crackers, maintenance/construction, moved cages, vet, visitors, and weather (Table 1). The most common occurring of these stressors were bellowing, maintenance/construction, and visitors.

Wildlife being held in captivity experiences many routine husbandry activities on a daily basis such as feeding and cleaning. Coping with being held in captivity, a place which is dramatically different to that which they are adapted to, is already challenging enough for wildlife [37]. When someone enters the enclosure of a wild animal, their natural instinct to escape is inhibited, and this can cause a rise in their stress response [38]. Some studies suggest that wildlife can habituate to routine husbandry activities through signalled predictability paired with temporal predictability by being able to predict and anticipate when events would occur [37]. There are no studies published to date to our knowledge that monitor habituation of routine husbandry activities in koalas, however there is one such study that shows that koalas can become increasingly vigilant when within a 5 m proximity to people [39]. In order to manage the potential stress of routine husbandry activities, only experienced staff are used by the Port Stephens Koala Hospital to enter koala enclosures. Furthermore, they are instructed not to bother the koalas, instead they are just to enter the enclosures, conduct routine husbandry activities, and leave.

Animal vocalisations occur in a variety of contexts, and usually bellowing in koalas occurs as a sexual advertisement call of males to females [40]. It is not unusual for male koalas to have been bellowing at the time of data collection in this study, as September to December coincides with the koala breeding season [41]. Previous studies have been able to detect considerable differences between koalas during the breeding season with a rise in glucocorticoids, compared to the non-breeding season [34].

Research shows that wildlife being held in captivity can adapt to noises heard on a regular basis, however the noise of maintenance/construction can be particularly stressful [42]. This is because the noise of maintenance/construction can be intense and often occurs unpredictably [42]. Several studies have been published that describe aversive responses of wildlife to maintenance/construction when being held in captivity, with examples including studies performed in snow leopards (*Panthera uncia*) [43], giant pandas (*Ailuropoda melanoleuca*) [44], and Hawaiian honeycreepers (*Drepanidinae* spp.) [45].

It has been well documented that visitors can elicit a stress response from wildlife being held in captivity [46], and this is especially so for koalas [47]. Some studies suggest that animals can habituate to visitors after a period of time [48], however most studies suggest that visitors in fact elicit a response in animals that results in a rise in glucocorticoids [46]. However, this does depend on the temperament of the species or an individual, and the behaviour of the visitors themselves [49].

## 5. Conclusions

The aim of this study was to quantify faecal and fur cortisol metabolites in wild rescued koalas undergoing wildlife rehabilitation. It was hypothesised that faecal glucocorticoids and fur glucocorticoids between koalas would differ based on individual responses to stressors. Statistically, there was a significant difference between all three koalas and their faecal cortisol responses, as well as their fur cortisol responses. Statistically for Maree and Solstice, there was a significant difference in their faecal cortisol response between days when a stressor was recorded, and days when a stressor was not recorded. However, statistically for Tai, this was not the case, as there was no significant difference in his faecal cortisol response between days when a stressor was recorded, and days when a stressor was not recorded. In summary, the hypothesis that faecal glucocorticoids and fur glucocorticoids between koalas will differ based on individual responses to stressors was true as a whole, but individually, this hypothesis was true for Maree and Solstice, but untrue for Tai.

This pilot study was restricted by the number of koalas available at the time of data collection, and as a result, there is a need for a larger and more detailed study. Furthermore, there is no current glucocorticoid baseline with which to compare the results of this study, and a gap remains for baseline glucocorticoid data for faecal and fur samples in wild koalas. As a result, measuring faecal and fur glucocorticoids is the first step in understanding how koalas undergoing wildlife rehabilitation respond to stressors.

## Figures and Tables

**Table 1:** Various stressors experienced by koalas within clinical care.

Stressor	Description
**Bellowing**	Refers to hearing one or more other koalas in the sanctuary bellowing
**Campers**	Refers to people staying at the campsite which neighbours the sanctuary
**Fire-Crackers**	Refers to hearing fire-crackers being released on the outskirts of the sanctuary
**Maintenance/Construction**	Refers to maintenance happening around the sanctuary, or construction happening on the outskirts of the sanctuary
**Moved Cages**	Refers to the koala being placed in a new enclosure
**Vet**	Refers to the koala being transported to a veterinary clinic for treatment or a check up
**Visitors**	Refers to tourists visiting the sanctuary
**Weather**	Refers to severe weather events such as storms or hail

## Data Availability

Data is contained within the article or supplementary material.

## Supplementary Materials

The following are available online at https://www.mdpi.com/article/10.3390/ani11102864/s1, Supplementary file 1.

## References

[1] Hundloe T., Hamilton C., Wilks L. (1997). Koalas and Tourism: An Economic Evaluation.

[2] Charalambous R., Narayan E. (2020). A 29-year retrospective analysis of koala rescues in New South Wales, Australia. PLoS ONE.

[3] Gonzalez-Astudillo V., Allavena R., McKinnon A., Larkin R., Henning J. (2017). Decline causes of koalas in south east Queensland, Australia: A 17-year retrospective study of mortality and morbidity. Sci. Rep..

[4] Cooper J., Cooper M.E. (2006). Ethical and legal implications of treating casualty wild animals. In Pract..

[5] Kwok A.B.C., Haering R., Travers S.K., Stathis P. (2021). Trends in wildlife rehabilitation rescues and animal fate across a six-year period in New South Wales, Australia. PLoS ONE.

[6] Orr B., Tribe A. (2018). Animal welfare implications of treating wildlife in Australian veterinary practices. Aust. Vet. J..

[7] Beehner J.C., Bergman T.J. (2017). The next step for stress research in primates: To identify relationships between glucocorticoid secretion and fitness. Horm. Behav..

[8] Denver R.J. (2009). Structural and functional evolution of vertebrate neuroendocrine stress systems. Ann. N. Y. Acad. Sci..

[9] Sapolsky R.M., Romero L.M., Munck A.U. (2000). How do glucocorticoids influence stress responses? Integrating permissive, suppressive, stimulatory and prepartive actions. Endocr. Rev..

[10] Romero L.M., Dickens M.J., Cyr N.E. (2009). The reactive scope model—A new model integrating homeostasis, allostasis, and stress. Horm. Behav..

[11] O’Connor T.M., O’Halloran D.J., Shanahan F. (2000). The stress response and the hypothalamic-pituitary-adrenal axis: From molecule to melancholia. QJM.

[12] Selye H. (1955). The Stress Concept. J. Chronic Dis..

[13] Wingfield J.C., Sapolsky R.M. (2003). Reproduction and resistance to stress: When and how. J. Neuroendocrinol..

[14] Hing S., Narayan E.J., Thompson R.C.A., Godfrey S.S. (2016). The relationship between physiological stress and wildlife disease: Consequences for health and conservation. Wildl. Res..

[15] Chrousos G.P. (2009). Stress and disorders of the stress system. Nat. Rev. Endocrinol..

[16] Karalis K., Sano H., Redwine J., Listwak S., Wilder R.L., Chrousos G.P. (1991). Autocrine or Paracrine Inflammatory Actions of Corticotropin-Releasing Hormone in Vivo. Science.

[17] Chrousos G.P. (1992). The concepts of stress and stress system disorders. Overview of physical and behavioral homeostasis. J. Am. Med. Assoc..

[18] Grogan L.F., Peel A.J., Kerlin D., Ellis W., Jones D., Hero J.M., McCallum H. (2018). Is disease a major causal factor in declines? An evidence framework and case study on koala chlamydiosis. Biol. Conserv..

[19] Keay J.M., Singh J., Gaunt M.C., Kaur T. (2006). Fecal Glucocorticoids and Their Metabolites as Indicators of Stress in Various Mammalian Species: A Literature Review. J. Zoo Wildl. Med..

[20] Sheriff M.J., Dantzer B., Delehanty B., Palme R., Boonstra R. (2011). Measuring stress in wildlife: Techniques for quantifying glucocorticoids. Oecologia.

[21] Narayan E., Hero J.M., Evans N., Nicolson V., Muccib A. (2012). Non-invasive evaluation of physiological stress hormone responses in a captive population of the greater bilby *Macrotis lagotis*. Endanger. Species Res..

[22] Narayan E., Vanderneut T. (2019). Physiological stress in rescued wild koalas are influenced by habitat demographics, environmental stressors, and clinical intervention. Front. Endocrinol..

[23] Narayan E.J., Webster K., Nicolson V., Mucci A., Hero J.M. (2013). Non-invasive evaluation of physiological stress in an iconic Australian marsupial: The koala (*Phascolarctos cinereus*). Gen. Comp. Endocrinol..

[24] Cork S.J. (1996). Optimal digestive strategies for arboreal herbivorous mammals in contrasting forest types: Why koalas and colobines are different. Aust. J. Ecol..

[25] Touma C., Palme R. (2005). Measuring fecal glucocorticoid metabolites in mammals and birds: The importance of validation. Ann. N. Y. Acad. Sci..

[26] Burnard C., Ralph C., Hynd P., Hocking-Edwards J., Tilbrook A. (2017). Hair cortisol and its potential value as a physiological measure of stress response in human and non-human animals. Anim. Prod. Sci..

[27] Woods J.G., Paetkau D., Lewis D., McLellan B.N., Proctor M., Strobeck C. (1999). Genetic tagging of free-ranging black and brown bears. Wildl. Soc. Bull..

[28] Charalambous R., Narayan E. (2019). Cortisol measurement in koala (*Phascolarctos cinereus*) fur. J. Vis. Exp..

[29] Macbeth B.J., Cattet M.R.L., Stenhouse G.B., Gibeau M.L., Janz D.M. (2010). Hair cortisol concentration as a noninvasive measure of long-term stress in free-ranging grizzly bears (*Ursus arctos*): Considerations with implications for other wildlife. Can. J. Zool..

[30] Bortolotti G.R., Marchant T.A., Blas J., German T. (2008). Corticosterone in Feathers Is a Long-Term, Integrated Measure of Avian Stress Physiology. Funct. Ecol..

[31] Wennig R. (2000). Potential problems with the interpretation of hair analysis results. Forensic Sci. Int..

[32] Santamaria F., Barlow C.K., Schlagloth R., Schittenhelm R.B., Palme R., Henning J. (2021). Identification of Koala (*Phascolarctos cinereus*) Faecal Cortisol Metabolites Using Liquid Chromatography-Mass Spectrometry and Enzyme Immunoassays. Metabolites.

[33] Palme R. (2019). Non-invasive measurement of glucocorticoids: Advances and problems. Physiol. Behav..

[34] Santamaria F., Palme R., Schlagloth R., Klobetz-Rassam E., Henning J. (2021). Seasonal Variations of Faecal Cortisol Metabolites in Koalas in South East Queensland. Animals.

[35] Lloyd J.K.F. (2017). Minimising Stress for Patients in the Veterinary Hospital: Why It Is Important and What Can Be Done about It. Vet. Sci..

[36] Fischer C.P., Romero L.M. (2019). Chronic captivity stress in wild animals is highly species-specific. Conserv. Physiol..

[37] Gottlieb D.H., Coleman K., McCowan B. (2013). The Effects of Predictability in Daily Husbandry Routines on Captive Rhesus Macaques (*Macaca mulatta*). Appl. Anim. Behav. Sci..

[38] Morgan K.N., Tromborg C.T. (2007). Sources of stress in captivity. Appl. Anim. Behav. Sci..

[39] Larsen M.J., Sherwen S.L., Rault J. (2014). Number of nearby visitors and noise level affect vigilance in captive koalas. Appl. Anim. Behav. Sci..

[40] Ellis W., Bercovitch F., FitzGibbon S., Roe P., Wimmer J., Melzer A., Wilson R. (2011). Koala bellows and their association with the spatial dynamics of free-ranging koalas. Behav. Ecol..

[41] Ballantyne K., Lisle A., Mucci A., Johnston S.D. (2015). Seasonal oestrous cycle activity of captive female koalas in south-east Queensland. Aust. Mammal..

[42] Jakob-Hoff R., Kingan M., Fenemore C., Schmid G., Cockrem J.F., Crackle A., Bemmel E.V., Connor R., Descovich K. (2019). Potential Impact of Construction Noise on Selected Zoo Animals. Animals.

[43] Sulser C.E., Steck B.L., Baur B. (2008). Effects of construction noise on behaviour of and exhibit use by Snow leopards *Uncia uncia* at Basel zoo. Int. Zoo Yearb..

[44] Powell D.M., Carlstead K., Tarou L.R., Brown J.L., Monfort S.L. (2006). Effects of construction noise on behavior and cortisol levels in a pair of captive giant pandas (*Ailuropoda melanoleuca*). Zoo Biol..

[45] Shepherdson D.J., Carlstead K.C., Wielebnowski N.C. (2004). Cross-institutional assessment of stress responses in zoo animals using longitudinal monitoring of faecal cortcoids and behaviour. Anim. Welf..

[46] Fernandez E.J., Tamborski M.A., Pickens S.R., Timberlake W. (2009). Animal–visitor interactions in the modern zoo: Conflicts and interventions. Appl. Anim. Behav. Sci..

[47] Webster K., Narayan E., De-Vos N. (2017). Fecal glucocorticoid metabolite response of captive koalas (*Phascolarctos cinereus*) to visitor encounters. Gen. Comp. Endocrinol..

[48] Margulis S.W., Hoyos C., Anderson M. (2003). Effect of felid activity on zoo visitor interest. Zoo Biol..

[49] Hosey G.R. (2000). Zoo animals and their human audiences: What is the visitor effect?. Anim. Welf..

